# Age at puberty and risk of asthma: A Mendelian randomisation study

**DOI:** 10.1371/journal.pmed.1002634

**Published:** 2018-08-07

**Authors:** Cosetta Minelli, Diana A. van der Plaat, Bénédicte Leynaert, Raquel Granell, Andre F. S. Amaral, Miguel Pereira, Osama Mahmoud, James Potts, Nuala A. Sheehan, Jack Bowden, John Thompson, Debbie Jarvis, George Davey Smith, John Henderson

**Affiliations:** 1 Population Health and Occupational Disease, National Heart and Lung Institute, Imperial College London, London, United Kingdom; 2 UMR 1152, INSERM, Paris, France; 3 UMR 1152, Université Paris Diderot, Paris, France; 4 MRC Integrative Epidemiology Unit, University of Bristol, Bristol, United Kingdom; 5 Population Health Sciences, University of Bristol, Bristol, United Kingdom; 6 Department of Health Sciences, University of Leicester, Leicester, United Kingdom; King's College London, UNITED KINGDOM

## Abstract

**Background:**

Observational studies on pubertal timing and asthma, mainly performed in females, have provided conflicting results about a possible association of early puberty with higher risk of adult asthma, possibly due to residual confounding. To overcome issues of confounding, we used Mendelian randomisation (MR), i.e., genetic variants were used as instrumental variables to estimate causal effects of early puberty on post-pubertal asthma in both females and males.

**Methods and findings:**

MR analyses were performed in UK Biobank on 243,316 women using 254 genetic variants for age at menarche, and on 192,067 men using 46 variants for age at voice breaking. Age at menarche, recorded in years, was categorised as early (<12), normal (12–14), or late (>14); age at voice breaking was recorded and analysed as early (younger than average), normal (about average age), or late (older than average). In females, we found evidence for a causal effect of pubertal timing on asthma, with an 8% increase in asthma risk for early menarche (odds ratio [OR] 1.08; 95% CI 1.04 to 1.12; *p =* 8.7 × 10^−5^) and an 8% decrease for late menarche (OR 0.92; 95% CI 0.89 to 0.97; *p =* 3.4 × 10^−4^), suggesting a continuous protective effect of increasing age at puberty. In males, we found very similar estimates of causal effects, although with wider confidence intervals (early voice breaking: OR 1.07; 95% CI 1.00 to 1.16; *p =* 0.06; late voice breaking: OR 0.93; 95% CI 0.87 to 0.99; *p =* 0.03). We detected only modest pleiotropy, and our findings showed robustness when different methods to account for pleiotropy were applied. BMI may either introduce pleiotropy or lie on the causal pathway; secondary analyses excluding variants associated with BMI yielded similar results to those of the main analyses. Our study relies on self-reported exposures and outcomes, which may have particularly affected the power of the analyses on age at voice breaking.

**Conclusions:**

This large MR study provides evidence for a causal detrimental effect of early puberty on asthma, and does not support previous observational findings of a U-shaped relationship between pubertal timing and asthma. Common biological or psychological mechanisms associated with early puberty might explain the similarity of our results in females and males, but further research is needed to investigate this. Taken together with evidence for other detrimental effects of early puberty on health, our study emphasises the need to further investigate and address the causes of the secular shift towards earlier puberty observed worldwide.

## Abstract 2

Using genetic instruments, Cosetta Minelli and colleagues probe the causal nature of associations between early puberty, late puberty, and late-onset asthma.

## Author summary

**Why was this study done?:**

- Worldwide there has been a shift towards earlier puberty over time, possibly due to changes in childhood lifestyle and levels of adiposity.
- Early puberty has been linked to an increased risk of asthma in women, but findings are inconsistent across studies, and little research has been done in men.
- There is some suggestion that late puberty might also increase the risk of asthma.
- Studies performed so far have some methodological limitations that make it difficult to distinguish true effects from spurious findings.

**What did the researchers do and find?:**

- We studied the effect of age at puberty on asthma in women and men using Mendelian randomisation, an approach that exploits genetic data to overcome some limitations of classical observational studies.
- From the UK Biobank study, we analysed data on approximately 240,000 women and approximately 190,000 men with available genetic data and self-reported information on asthma and age at puberty (menarche for women and voice breaking for men).
- We found that women and men with early puberty had an increased risk of asthma of 8% and 7%, respectively.
- We found no evidence of a detrimental effect of late puberty on asthma.

**What do these findings mean?:**

- These findings, together with suggestions of other detrimental effects of early puberty on health, emphasise the importance of evaluating the overall evidence on health effects of early puberty.
- The similarity of findings in females and males might be explained by common biological or psychological factors related to early puberty, but further research is needed to investigate this.

## Introduction

Asthma is one of the most common chronic diseases in children and adults, and its prevalence has been increasing worldwide [1]. Multiple risk factors for asthma have been proposed, but one of the few consistent observations is sex differences in disease prevalence. Understanding the reason for these differences could lead to the identification of modifiable factors that could be targeted by preventive measures [2].

Sex differences in asthma prevalence are age dependent, with a switch from male predominance in childhood to female predominance from adolescence onwards [3,4]. It has been hypothesized that the higher prevalence of post-pubertal asthma in females, with higher incidence and lower remission rates [4], might be due to a pathogenetic role of female sex hormones [3,5]. This is further supported by observational findings of an association of early menarche with increased risk of post-pubertal asthma [6]. In addition to the hypothesis of sex hormones mediating the effect of early menarche on asthma, another proposed mechanism is through obesity: Early menarche would increase the risk of adult obesity, which would in turn affect asthma, possibly through an effect of higher leptin levels on the immune system and airway inflammation, although it could be the other way round, with higher childhood BMI and leptin increasing the risk of early menarche, which would increase the risk of asthma [7,8]. Despite the plausibility that the observed association of early menarche with asthma may represent a causal effect, this cannot be concluded based on the available evidence, and, if there is a causal effect, it is unclear what its magnitude may be. A meta-analysis of 7 observational studies suggested a 37% increase in asthma risk for women with menarche before the age of 12 years, but there was substantial heterogeneity in results across studies [9]. The meta-analysis was mainly based on young study populations, and an observational study in UK Biobank including women aged 40 to 69 years suggested a smaller increase of 6% in asthma risk after adjustment for confounders [10]. The same study also showed an association in males, with an 11% increase in risk associated with early voice breaking, a marker of male puberty, and it suggested a U-shaped relationship in females and males, with an increased risk of asthma associated with both early and late puberty [10]. More recently, a prospective study in women up to the age of 60 years suggested no association after extensive adjustment for confounders [11]. The possibility of residual confounding by unmeasured factors, including intrauterine exposures as well as childhood diet, lifestyle and socioeconomic status, limits the interpretation of these findings and may explain inconsistencies across studies. There is also a potential problem of reverse causation, as there are some data suggesting that girls with childhood asthma might have earlier menarche [12]. This could generate spurious associations, particularly if study participants with pre-pubertal asthma were not excluded from the analyses due to poor recall of their childhood symptoms.

The Mendelian randomisation (MR) approach applied here uses genetic variants known to affect age at puberty as proxies (instrumental variables) to derive estimates of the effect of age at puberty on asthma [13]. As genes are randomly allocated at the time of conception, genetic associations are not affected by typical confounding factors or reverse causation, and MR can provide indirect evidence for a causal effect if its underlying assumptions hold [13,14]. Here we describe a large MR study in UK Biobank aimed at estimating the causal effect of age at puberty on the risk of post-pubertal asthma in females and males.

## Methods

The UK Biobank is a prospective study of about 500,000 adults aged 40–69 years recruited in 2006–2010 across 22 centres, aimed at identifying causes of chronic disease in middle and old age [15]. About 95% of the participants are white. Data on doctor-diagnosed asthma and age at onset (AAO), age at menarche in females, and age at voice breaking in males are all based on self-reports from questionnaires. The specific questions used to define the variables used are reported in Table 1, together with the UK Biobank data-field number for access to further information on measurement procedures through the UK Biobank webpage (http://biobank.ctsu.ox.ac.uk/crystal/search.cgi). While age at menarche was recorded in years, age at voice breaking was recorded as a 3-level categorical variable (at a younger, average, or older age compared to peers). In order to increase comparability with the findings in males, we categorised age at menarche in females as early (<12 years), normal (12 to 14), or late (>14), using “normal” as the reference. This also allowed the investigation of possible non-linear associations, such as U-shaped relationships. In females, we defined post-pubertal asthma as asthma occurring after menarche; in males, this was defined as asthma occurring after the age of 15 years. Participants with pre-pubertal asthma were excluded from the analyses.

**Table 1:** Characteristics of the study population, for females and males.

Variable	Females (*N =* 243,316)	Males (*N =* 192,067)	Definition
**Age (years)**	56.4 (8.0)	56.8 (8.2)	Age at assessment [data-field 21003]
**Age at menarche (years)**	13.0 (1.6)		Self-reported (“How old were you when your periods started?”) [data-field 2714]
Early (<12)	48,840 (20.1)		
Normal (12–14)	153,727 (63.2)		
Late (>14)	40,749 (16.7)		
**Voice breaking**			Self-reported (“When did your voice break?”) [data-field 2385]
Early (younger than average)		8,353 (4.3)	
Normal (about average age)		172,604 (89.9)	
Late (older than average)		11,110 (5.8)	
**BMI (kg/m^2^)**	27.0 (5.1)	27.8 (4.2)	Calculated from measured height and weight [data-field 21001]
**Asthma**	22,295 (9.2)	12,066 (6.3)	Self-reported doctor-diagnosed asthma (“Has a doctor ever told you that you have asthma?”) [data-field 6152 (all participants) + data-field 22127 (subset from online occupational follow-up)]^1^
**Asthma AAO (years)**	39.7 (13.4)	41.0 (13.1)	Self-reported (“What was your age when the asthma was first diagnosed?”) [data-field 3786]

Reported are mean (SD) for continuous variables and *N* (%) for categorical variables. Only post-pubertal asthma is considered. Data-field: UK Biobank variable’s identifier (link to further information through website: http://biobank.ctsu.ox.ac.uk/crystal/search.cgi). Asthma and age at onset (AAO) refer to post-pubertal asthma.

^1^Asthma was considered present if there was a positive answer in either of the 2 data-fields.

### Choice of instruments for MR analyses

We chose MR instruments for age at menarche based on findings from the large genome-wide association (GWA) meta-analysis by Day et al. [16], which included 329,345 women of European ancestry from the ReproGen consortium (40 studies, *N =* 179,117), 23andMe (*N =* 76,831), and UK Biobank (*N =* 73,397). In all, 389 independent single nucleotide polymorphisms (SNPs) were identified at a genome-wide significant level (*p <* 5 × 10^−8^), explaining overall 7.4% of the population variance in age at menarche. Of the reported 389 SNPs, 372 were available (or had a proxy, linkage disequilibrium *r*^2^ > 0.7) in UK Biobank.

The instrument strength of each SNP for both early and late menarche was assessed using the F statistic, a function of the magnitude and precision of the SNP’s effect on menarche [17]. To avoid bias associated with the use of weak instruments, we excluded SNPs with a low F statistic in UK Biobank, using the common threshold of 10 [18]. This left us with 206 and 151 SNPs (254 in total) as instruments for early and late menarche, respectively; F statistic values varied from 10.1 to 382.5 for early menarche and from 10.0 to 150.0 for late menarche (S1 Table). All SNPs had good imputation quality (info score > 0.8).

In the GWA meta-analysis by Day et al. [16], 127 of the 389 age-at-menarche SNPs were also associated in the same direction with age at voice breaking at nominal significance level; of these, only 40 were strong instruments (F statistic > 10) for either early or late puberty in males. In order to increase our number of instruments, we searched the literature for additional genetic studies on age at voice breaking, using the NHGRI GWAS Catalog [19] and HuGE Navigator [20], as well as crosschecking of references. We identified 3 other GWA studies [21–23], 2 of which added new independent SNPs, the studies by Day et al. [22] and by Pickrell et al. [23] (S2 Table). After combining all SNPs and excluding weak instruments, 24 and 37 SNPs (46 in total) remained for the MR analyses of early and late voice breaking, respectively. Their F statistic values varied from 10.2 to 52.4 for early voice breaking and from 10.8 to 101.6 for late voice breaking (S2 Table). All SNPs had good imputation quality (info score > 0.9). All 46 SNPs were independent, except for 3 pairs of correlated SNPs (linkage disequilibrium *r*^2^ of 0.3, 0.6, and 0.6). As a post hoc analysis to investigate the impact of the correlation between these SNPs on our MR results, we used the method by Burgess et al., which accounts for the correlation between instruments [24].

Details on genotyping and imputation methods and quality control procedures in UK Biobank are available elsewhere [25].

### MR methods

In 1-sample MR investigations (where the genetic associations with both risk factor and outcome are estimated within the same study), the standard MR approach is the 2-stage least squares (2SLS) method: the risk factor is regressed jointly on all SNPs, and the outcome is regressed on the genetically predicted values of the risk factor from the first regression [26]. As this method does not account for pleiotropy (where SNPs chosen as instruments for the risk factor affect the outcome through additional independent pathways [14]) and we cannot rule out the possibility of pleiotropic effects for some of our SNPs, we used methods developed for 2-sample MR. Here estimates of the genetic associations with risk factor and outcome are obtained separately for each SNP. If all SNPs are valid instruments (e.g., no pleiotropy) and linear models with no interactions faithfully describe the SNP–risk factor and risk factor–outcome relationships [27], the individual MR estimates of causal effect will vary only by chance, with no between-instrument heterogeneity [28]. For each SNP, the association with early or late menarche/voice breaking (G–X, expressed as log odds ratio [OR]) and the association with asthma (G–Y, log OR) were estimated using multiple logistic regression adjusted for assessment centre, first 10 ancestry principal components, and genotyping array. It should be noted that with binary variables the linearity assumption is strictly violated [29], but in practice the magnitude of the bias has been shown to be negligible [30].

An MR estimate was obtained for each SNP using a Wald estimator (ratio of G–Y over G–X), with standard error derived using the Delta method [31], and individual MR estimates were pooled using inverse-variance weighted (IVW) fixed-effect meta-analysis [32]. The pooled MR estimate is asymptotically equal to the MR estimate obtained with the 2SLS method [32], which we also performed as a sensitivity analysis. Since the IVW fixed-effect meta-analysis method assumes no pleiotropy, we performed secondary analyses using approaches based on different assumptions about pleiotropy across SNPs: (1) IVW random-effect meta-analysis, assuming random pleiotropic effects (i.e., they cancel out) [33]; (2) MR-Egger regression with penalised weights, assuming directional pleiotropic effects [34]; and (3) weighted median analysis, assuming that at least 50% of the SNPs are valid instruments (but with no assumption about direction of pleiotropic effects) [35].

Some of the genetic variants associated with pubertal timing are also associated with BMI [16], and this may complicate the interpretation of the results given that BMI could either introduce pleiotropy (i.e., affecting asthma independently from age at puberty) or lie on the causal pathway. In secondary analyses we therefore excluded SNPs associated with BMI from our instruments. We identified SNPs associated with BMI, overweight, or obesity in previous GWA studies from PhenoScanner, a database of publicly available GWA findings (http://www.phenoscanner.medschl.cam.ac.uk/phenoscanner) [36], using a significance threshold corrected for multiple testing, *p <* 2.0 × 10^−4^ (0.05/254) for menarche SNPs and *p <* 1.1 × 10^−3^ (0.05/46) for voice breaking SNPs.

To provide further support for the absence of pleiotropy, we used pre-pubertal asthma as a “negative control” outcome [37]. If our instruments affect asthma solely through age at menarche/voice breaking, we expect to find no effect on asthma developed before puberty.

There was no formal pre-specified protocol for this study; all the analyses described above were decided a priori, and we performed additional post hoc analyses suggested by the reviewers. In particular, sensitivity analyses were carried out to assess the robustness of the results to the use of different AAO cutoffs for post-pubertal asthma definition in males, and to the presence of some correlation between genetic variants. Subgroup analyses were also performed to investigate the possibility that the effect of age at puberty on asthma might be modified by whether or not one is overweight.

All analyses were performed using Stata 15 (StataCorp).

## Results

The characteristics of the study population are summarised in Table 1. We included 243,316 women and 192,067 men with available genetic data and information on age at puberty, asthma and AAO; post-pubertal asthma was present in 22,295 (9.2%) women and 12,066 (6.3%) men. Asthma prevalence by category of age at puberty was 10.4% for early, 8.9% for normal, and 8.8% for late menarche in females, and 7.5% for early, 6.2% for normal, and 7.1% for late age at voice breaking in males.

### MR analyses

Detailed results from all MR analyses are reported in S3 and S4 Tables, for females and males, respectively. In females, we found evidence for a detrimental causal effect of early menarche, with an OR of 1.08 (95% CI 1.04 to 1.12; *p =* 8.7 × 10^−5^), and for a protective causal effect of late menarche, with an OR of 0.92 (95% CI 0.89 to 0.97; *p =* 3.4 × 10^−4^). This suggests a continuous protective effect of increasing age at puberty, and does not support observational evidence of a U-shaped relationship. When we repeated the analysis using age at menarche as a continuous variable (332 SNPs with F > 10 as instruments), we found an OR of 1.06 (95% CI 1.03 to 1.11; *p =* 1.6 × 10^−4^) per year decrease in age at menarche. In males, we found an OR of 1.07 (95% CI 1.00 to 1.16; *p =* 0.064) for early and 0.93 (95% CI 0.87 to 0.99; *p =* 0.029) for late voice breaking. The results remained the same in sensitivity analyses allowing for the correlation between SNPs, and became slightly stronger in sensitivity analyses using higher age cutoffs for asthma AAO to define post-pubertal asthma, specifically AAO >16 and >17 years (S4 Table). These findings in males are very similar to those in females, despite the much wider confidence intervals reflecting the lower power of the analyses (fewer instruments available for age at voice breaking and possibly larger measurement error). The use of a 1-sample 2SLS approach gave similar results in both females and males (S3 and S4 Tables).

**Fig 1:**
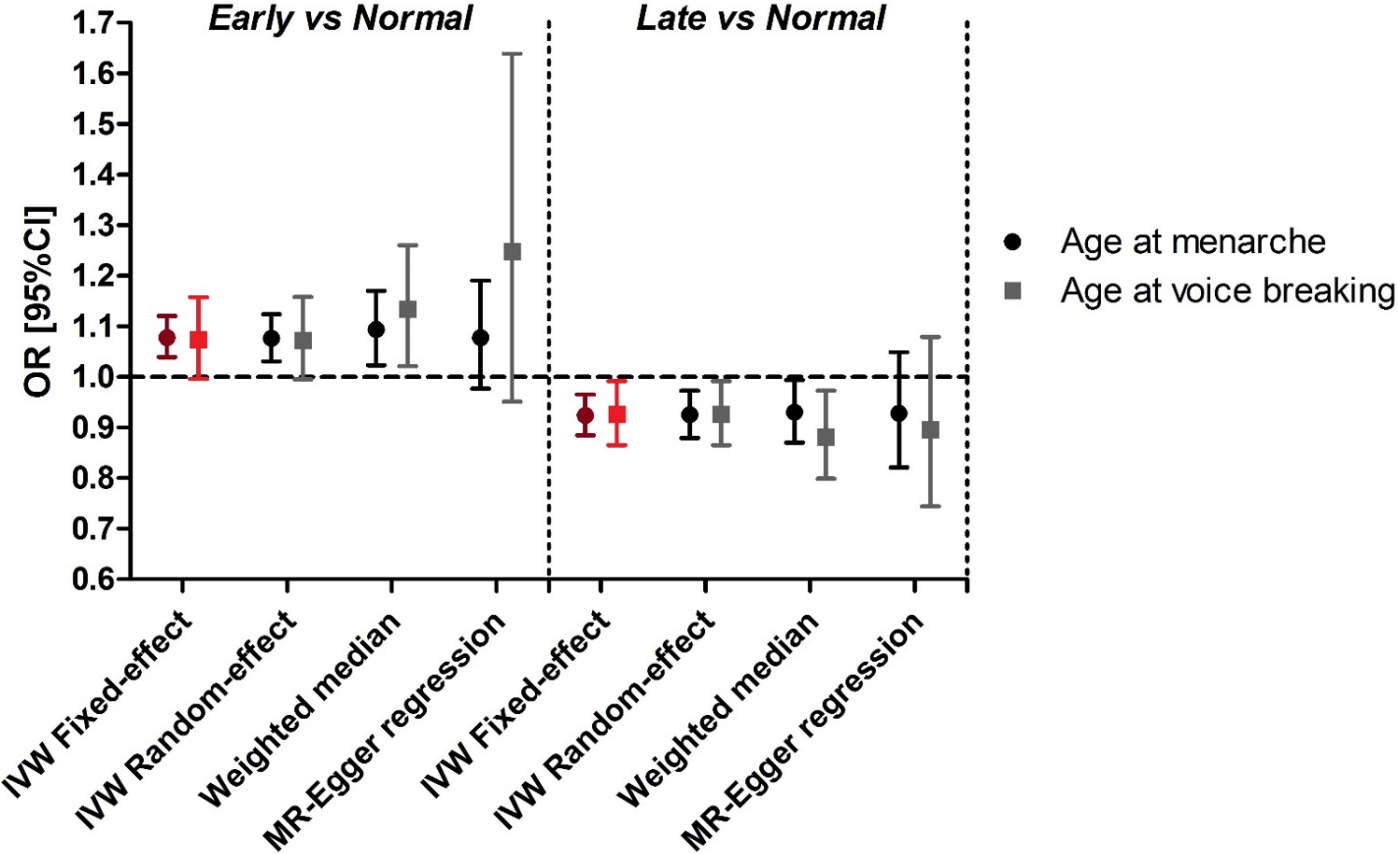
Estimates across different MR methods of the effects of early and late puberty on asthma risk in females (menarche) and males (voice breaking). In red: main analysis. IVW, inverse-variance weighted; MR, Mendelian randomisation.

We found only modest between-instrument heterogeneity in females (*I*^2^ of 16% and 20% for early and late puberty, respectively; S3 Table) and little evidence of heterogeneity in males (*I*^2^ of 2% and 0%, respectively; S4 Table). The results of the analyses controlling for pleiotropy were consistent with those of the main analyses (Fig 1), with differences in the precision of the estimates in line with expected differences in statistical power [33,35]. MR-Egger regression in males suggested stronger effects compared to the main analysis, particularly for early puberty (Fig 1), but the confidence intervals were wide as the analysis had limited power. This low power is due to the low number of SNPs and their relatively similar instrument strengths (MR-Egger regression works best when there is a large spread of strengths [38]).

Secondary analyses with exclusion of SNPs associated with BMI (14 and 12 excluded for early and late menarche; 5 and 7 excluded for early and late voice breaking) gave similar results to those of the main analyses for both females and males (S3 and S4 Tables).

As the percentages of females in the early and late menarche categories were substantially higher than the percentages of males in the corresponding categories (Table 1), we repeated the analyses using more stringent cutoffs for age at menarche. Using a cutoff of <11 instead of <12 years for early and >15 instead of >14 years for late menarche, the percentages were very similar to those in males, 4.6% and 5.8% for early and late menarche, respectively. The results were in line with those of the main analyses, with an OR of 1.06 (95% CI 1.02 to 1.09; *p =* 6.4 × 10^−4^) for early and 0.95 (95% CI 0.91 to 0.98; *p =* 0.002) for late menarche.

The MR analyses on pre-pubertal asthma, used as a negative control outcome, suggested no effect for either age at menarche or age at voice breaking, further reassuring against effects of our genetic instruments on asthma independent from pubertal timing.

Finally, we repeated the analyses separately by BMI group, with low/high BMI defined as below/above the median (<26/≥26 kg/m^2^ in females; <27/≥27 kg/m^2^ in males), to investigate possible effect modification due to whether or not participants are overweight. BMI data refer to the participants’ BMI at the time of recruitment into UK Biobank (Table 1). In females, these analyses (S3 Table) showed effect estimates for early and late menarche consistent with the main analyses in the high BMI group, and no evidence for effects of early or late menarche in the low BMI group, but there was no statistical evidence of interaction (*p*-values of 0.23 and 0.67 for tests of the difference in estimates between BMI groups for early and late menarche, respectively). In males, there was no evidence of effect modification (S4 Table), as expected given the very low statistical power.

## Discussion

This MR study provides evidence for a detrimental causal effect of earlier age at puberty on the risk of developing asthma later in life. In females, we estimated an 8% increase in asthma risk for early menarche (<12 years) and an 8% decrease for late menarche (>14 years). A very similar pattern was observed in males, with a 7% increase in risk for early puberty and a 7% decrease for late puberty, although the evidence was weaker than in females due to the much lower statistical power. Previous observational studies on pubertal timing and asthma, mainly performed in females, provided conflicting results, possibly due to residual confounding, reverse causation, or inappropriate adjustment for factors that lie on the causal pathway. This study adds to current knowledge by providing evidence for a causal effect of early puberty on asthma in both females and males, and by clarifying the pattern of the relationship between pubertal timing and asthma. Previous observational analyses in UK Biobank [10] suggested that there could be a U-shaped relationship (both early and late puberty would be detrimental), but our MR study does not support this and suggests a continuous protective effect of increasing age at puberty. The discrepancy may be due to confounding of the observational findings by factors associated with both delayed puberty and increased risk of asthma.

The results of our post hoc analysis by BMI group are compatible with the possibility of a stronger effect of age at puberty on asthma in overweight females, but these results have major limitations that make it difficult to draw conclusions. Stratifying by BMI might introduce collider bias, whereby conditioning on a common effect of exposure and outcome generates a spurious association between the two [39]; BMI is indeed affected by some of the puberty SNPs and might also be affected by asthma through medication side effects or reduced physical exercise. Moreover, the power of these analyses in females is low, in particular to detect interaction, and even more so in males. Finally, in our analyses we could only use adult BMI; although childhood obesity is likely to track to obesity in adulthood, the inability to disentangle the two makes these analyses difficult to interpret. Further research using longitudinal data is needed to investigate a possible role of obesity in modifying the effect of age at puberty on asthma.

The similarity of effect estimates in females and males provides insight into possible underlying mechanisms. Previously reported associations of early menarche with asthma have been hypothesized to be explained by earlier and greater cumulative exposure to female sex hormones such as oestrogens or an unbalanced oestrogen/progesterone ratio [5,9], although experimental evidence in mice does not support this explanation [40]. A role for female sex hormones has also been suggested by a reported association between pregnancy, characterised by increased levels of progesterone and oestrogens, and asthma development [41], and by decreased incidence of asthma after natural menopause [42,43]. The similarity of our results in both sexes suggests that early menarche may affect asthma through mechanisms that go beyond female sex hormones. Other factors accompanying early puberty in both females and males could be responsible, including biological but also psychological factors, such as depression, anxiety, and psychosomatic symptoms [44]. Further investigation of these aspects is important to identify modifiable factors that mediate the effects of early puberty on asthma and other adverse health outcomes.

The effect estimates found in our large study are of modest magnitude and therefore of limited clinical relevance to an individual, but they have important implications at a population level. In Europe, age at menarche decreased by 2 to 3 months per decade between 1790 and 1980 [45] and continued to decrease at a lower rate in the following decades [46], with similar trends observed worldwide. Although the reasons for such a shift are not fully understood, there is evidence suggesting a role for changing childhood risk factors, including diet and obesity, psychological stress, and environmental exposures [47]. Childhood obesity is likely the strongest potentially preventable factor, and currently represents a global epidemic, with half of the world’s population predicted to be overweight or obese by 2030 [48]. Other environmental risk factors for early puberty warrant further investigation, including endocrine-disrupting chemicals found in many household products that are hypothesized to have transgenerational epigenetic effects [49]. Observational evidence, particularly in females, has suggested detrimental effects of early puberty on other health outcomes including lung function, BMI, cardiometabolic traits, cardiovascular disease, and cancer [10,50–55], with recent support from MR analyses [16,22,56,57]. Taken together with this evidence, our findings emphasise the importance of investigating modifiable risk factors for early puberty with the aim of reversing the secular shift towards earlier puberty observed worldwide.

### Strengths and weaknesses

MR is a valuable tool to assess causality in epidemiology as it is not affected by the classical confounding factors or reverse causation typical of observational studies. However, this technique is at its most reliable and transparent in the absence of pleiotropy, i.e., in this case, where the genetic instruments do not have direct effects on asthma independent of age at puberty [14]. As the risk of pleiotropy is high in MR investigations where many SNPs of uncertain biological function are included, we thoroughly investigated its potential impact on our findings. Obesity was identified a priori as a potential source of pleiotropic effects given its genetic overlap with pubertal timing, and in fact around 7% of our instruments for age at puberty were also associated with BMI. In practice, what role BMI might play in our study is unclear, as it could induce “horizontal” pleiotropy by affecting asthma independently from puberty, but it could also lie on the causal pathway either preceding puberty (high childhood BMI → early puberty → high asthma risk) or mediating its effects (early puberty → high adult BMI → high asthma risk), which is referred to as “vertical pleiotropy” [58]. Only horizontal pleiotropy would affect the validity of our MR findings. When excluding SNPs associated with BMI, we found very similar results to those of the main analyses. Pleiotropy could act through mechanisms other than obesity. We therefore investigated the likely magnitude of pleiotropy by assessing the heterogeneity among causal estimates from the individual SNPs (no heterogeneity if all valid instruments [28]), and found evidence of only modest heterogeneity. This was supported by the consistency in the findings from 3 different methods to adjust for pleiotropy (IVW random-effect, weighted median, and MR-Egger regression) with those of the main analyses. Finally, we repeated our analyses using pre-pubertal asthma as a negative control outcome, and the null results provided further reassurance against pleiotropic mechanisms independent from pubertal timing.

We used MR methods developed to investigate and adjust for pleiotropy in 2-sample MR, where estimates of the association of each SNP with risk factor and outcome are derived from separate studies, and the 2 estimates are assumed independent. The behaviour of these methods has yet to be studied in the 1-sample context, where independence of the 2 estimates is violated. However, the consistency of our findings with those derived from the 1-sample 2SLS method provides reassurance.

Our study is based on self-reported information on doctor-diagnosed asthma, age at menarche, and age at voice breaking, which introduces measurement error and potentially recall bias. However, since it is unlikely that the error in recalling age at puberty would correlate with the reporting of asthma or asthma AAO, and vice versa, the measurement error is likely to be random and only result in reduced power. Voice breaking represents the drop in the pitch of speech due to changes in vocal chords and larynx cartilage in response to androgen exposure during late puberty [59]. Despite having been suggested as a good marker of pubertal timing in males when assessed prospectively [60], age at voice breaking is much harder to recall in adult cross-sectional studies compared with the more clear-cut milestone of menarche in females, and may be subject to greater measurement error. This is a general problem and partly explains the limited knowledge about risk factors and effects of early puberty in males, for which more evidence from longitudinal birth cohort studies is needed. Another limitation is that we based the choice of MR instruments for age at menarche and, partly, age at voice breaking on findings from the GWA meta-analysis by Day et al. [16], of which UK Biobank genetic data (first release only) represented about 20% of the total sample. However, any overestimation of the SNP–age at puberty associations resulting from this would have pulled our MR estimates towards the null, therefore leading to underestimation of the true causal effects rather than to false positive results. Finally, findings from observational studies might be biased by “cohort effects” if individuals from younger generations are at different risk of both early (or late) puberty and asthma compared with older generations. Despite the secular trends of decreasing age at puberty [45] and increasing prevalence of asthma [1], this type of confounding would not bias the results of our MR study, unless the secular trends also affect the genetic variants used as instruments for age at puberty, which is unlikely.

In conclusion, we provide evidence for a causal detrimental effect of early puberty on asthma. Further studies are needed to investigate the mechanisms underlying this effect, and the similarity of our findings in females and males suggests a possible role for common biological or psychological factors accompanying early pubertal development. Taken together with evidence for other detrimental effects of early puberty on health, our study emphasises the need to investigate and address causes of the secular shift towards earlier puberty observed worldwide.

## Supporting information

S1 Table Data for the MR analyses of age at menarche in females. (XLSX)

S2 Table Data for the MR analyses of age at voice breaking in males. (XLSX)

S3 Table Results of main and secondary MR analyses of age at menarche in females. (XLSX)

S4 Table Results of main and secondary MR analyses of age at voice breaking in males. (XLSX)

## Abbreviations

2SLS: 2-stage least squares
AAO: age at onset
GWA: genome-wide association
IVW: inverse-variance weighted
MR: Mendelian randomisation
OR: odds ratio
SNP: single nucleotide polymorphism
